# The Value of Cancer Immunotherapy Summit at the 2016 Society for Immunotherapy of Cancer 31^st^ anniversary annual meeting

**DOI:** 10.1186/s40425-017-0241-6

**Published:** 2017-04-18

**Authors:** Howard L. Kaufman, Michael B. Atkins, Adam P. Dicker, Heather S. Jim, Louis P. Garrison, Roy S. Herbst, William T. McGivney, Steven Silverstein, Jon M. Wigginton, Peter P. Yu

**Affiliations:** 1 Rutgers Cancer Institute of New Jersey, New Brunswick, NJ 08901 USA; 2 Georgetown-Lombardi Comprehensive Cancer Center, Washington, DC 20016 USA; 3 Thomas Jefferson University, Philadelphia, PA 19107 USA; 4 H. Lee Moffitt Cancer Center & Research Institute, Tampa, FL 33612 USA; 5 University of Washington School of Pharmacy, Seattle, WA 98195 USA; 6 Yale School of Medicine, New Haven, CT 06510 USA; 7 McGivney Global Advisors, 130 W Lancaster Ave, Suite 301, Wayne, PA 19087 USA; 8 Melanoma Research Foundation, Woodcliff Lake, NJ 07077 USA; 9 MacroGenics, Inc., Rockville, MD 20850 USA; 10 Hartford HealthCare, Memorial Sloan Kettering Cancer Alliance, Hartford, CT 06102 USA

**Keywords:** Cancer immunotherapy, Value, Cost, Summit, Biomarkers, Patient-reported outcomes

## Abstract

As healthcare costs continue to rise, there has been great interest in understanding and defining the value of current therapeutic strategies for the treatment of cancer. Cancer immunotherapy has emerged as a clinically beneficial alternative to conventional therapies for a variety of malignancies. Characterized by broad clinical activity, durable response rates, distinct side effects, and unique response kinetics, immune-based agents are vastly different compared with traditional cytotoxic or targeted therapies. To date, however, value assessments in oncology have not focused on the unique aspects of cancer immunotherapy, which has resulted in a lack of understanding of the true value of these therapies. Therefore, the Society for Immunotherapy of Cancer (SITC) convened key stakeholders to address the critical issues that define the value of cancer immunotherapy in National Harbor, Maryland on November 13, 2016. Organized in collaboration with the American Society for Clinical Oncology (ASCO) and with over 1500 registrants, this Value of Cancer Immunotherapy Summit united research scientists, academic physicians, industry professionals, health economists, third-party payers, and patients to discuss critical issues surrounding the value framework for cancer immunotherapy. This half-day summit addressed the current landscape of cancer therapy value models, economic outcomes, the current status of predictive biomarkers, as well as presentations from third-party payers, industry representatives, patient outcome experts, and patient advocacy groups to gain their perspectives on the value of cancer immunotherapy. Here, we summarize the presentations and the dominant themes from this symposium, with the intention of providing insight on future directions and to develop recommendations to better define the value of cancer immunotherapy for patients with cancer.

## Introduction

According to projections from the Centers for Medicare and Medicaid Services (CMS), National Healthcare Expenditures (NHE) are expected to grow at a rate of 5.8% annually, accounting for 19.6% of the national gross domestic product by 2024 [1]. Although it currently represents a fraction of the overall healthcare expenditures, the cost of cancer care is one of the fastest growing areas of healthcare-related spending in the U.S. Globally, in 2015 the costs of oncology therapeutics and supportive care increased 11.5% from 2010 to $107 billion [2]. Projections that incorporate trends in incidence, survival, oncology practice patterns, and cost of cancer therapeutics estimate that the total cost of cancer care in the U.S. will rise to $173 billion in 2020 [3]. Although this rise in cost is due to several factors, including an increase in the demand for oncology care by an aging population, cancer has been historically rated as one of the mostly costly medical conditions to treat in adults, second only to heart conditions [4]. Combined with lost income due to disease symptoms and the debilitating side effects from treatment, the financial burden that patients with cancer face can severely reduce their quality of life and may affect their decisions to continue therapy. In recent years, the dramatic rise in the cost of treating cancer has been a subject of intense discussion and debate among members of the oncology healthcare community. There has, therefore, been a nationwide push to evaluate therapeutic strategies based on their overall value, which takes into account measures beyond financial costs, including expected clinical outcomes, potential side effects, and impact on patients’ quality of life. In addition, other stakeholders may include academic institutions, which value actionable research as part of their core mission, and investors/shareholders may value healthcare progress and/or cost savings.

In the last decade, cancer immunotherapies have profoundly changed the therapeutic landscape for cancer patients by providing a clinically beneficial alternative to conventional treatments. Recent U.S. Food and Drug Administration (FDA) approvals and the rapid expansion of indications for existing agents have made immunotherapies accessible to patients with a variety of malignancies, including melanoma, hematologic malignancies, non-small cell lung cancer (NSCLC), prostate cancer, kidney cancer, bladder cancer, and head and neck cancer [5]. With additional FDA approvals of new therapeutics on the horizon and drug combination approaches as well as adjuvant/neoadjuvant strategies in clinical trials, cancer immunotherapies are expected to significantly impact the current standard of care in the coming years. Because these agents are based on the ability of the immune system to recognize and eliminate cancer, immunotherapies have been associated with broad clinical activity, durable responses, clinically challenging but manageable side effects, and response kinetics that are unique compared with conventional cytotoxic and targeted therapies. However, previous reports and value frameworks centered on the value of cancer care have not taken the unique aspects of cancer immunotherapies into consideration, which has led to uncertainty about the true value of this therapeutic modality.

In order to initiate this dialogue and address the critical issues involved in the discussion on the value of immune-based agents, SITC convened a Value of Cancer Immunotherapy Summit on the final day of the SITC 31^st^ Anniversary Annual Meeting in Bethesda, Maryland on November 13, 2016. Organized in collaboration with ASCO, the summit included speakers representing a wide range of expertise in tumor immunology, academic medicine, health economics, payer community, pharmaceutical industry, patient outcomes, and patient advocacy. The program concluded with an extended open panel discussion as well as a question and answer session with audience participation in order to further define key issues specific to immunotherapy and drive priorities as well as recommendations to better define the value of cancer immunotherapy. This meeting report highlights key aspects of each presentation and covers the main topics discussed during this half-day program, with the intention to provide a synopsis of the meeting and to inform on future directions for this initiative.

## Meeting report

### Session I: Current landscape of cancer therapy value models, economic outcomes, and the patient perspective

#### Evaluation of current value models

In the opening presentation of the Summit, Dr. Peter P. Yu introduced key concepts in the value discussion and presented a detailed evaluation of current frameworks to assess the value of cancer treatment. Dr. Yu began his presentation with an overview of the concept of value in healthcare, which can be defined as the incremental improvement in net health outcomes divided by total financial costs. In this equation of therapeutic value, net health outcomes are assessed based on their ability to optimize patient health, which requires assessing both gains in health due to disease control and reductions in health due to toxicities of therapy. Consequently, more recent initiatives to measure health outcomes have included the benefits of palliation of disease related symptoms as well as short and long term side effects that negatively impact patient health. For example, the International Consortium for Health Outcomes Measurements (ICHOM) has recently developed standardized outcomes measures for colon and prostate cancer, which include traditional measures such as disease free and overall survival and additional measures, such as quality of life, side effects or complications of treatment, and patient-reported metrics of health [6].

Dr. Yu then presented considerations for defining the financial cost of cancer therapeutics. Although there is agreement among the healthcare community that the rising cost of cancer care is not sustainable, the conversation is less consistent on what is meant by cost of care. The financial costs associated with drugs are defined differently depending upon the perspective of the end-user. The CMS structures its payment models based on the average sales price (ASP), which is based on the transaction cost between the drug manufacturer and the drug distributor. Healthcare systems and providers define costs by what is paid to the drug distributor and patients define costs based on their copays, co-insurance, and deductible obligation. Employers define costs in terms of the cost of providing employee insurance benefits, and pharmaceutical companies consider their research and development costs to bring drugs to the market. Thus, each of these perspectives must be considered and will impact the assessment of overall financial costs when determining value.

Following the introduction of the key concepts in the discussion on value, Dr. Yu described the current value frameworks that have been developed to assess cancer treatments, including ASCO’s Value Framework, the European Society of Medical Oncology’s Magnitude of Clinical Benefit Scale (ESMO-MCBS), the National Comprehensive Cancer Network (NCCN) Evidence Blocks, the Memorial Sloan Kettering Cancer Center (MSKCC)’s Drug Abacus, and the Institute for Clinical and Economic Review (ICER) Value Assessment Framework. In presenting an overview of each model, Dr. Yu highlighted the overall objectives, unique aspects, and intended audiences of each (Table 1). As a final example, the World Health Organization (WHO) Essential Medications List, a list of drugs every country should provide for their citizens, was presented. The update to the previous Essential Medication List defined a new value framework to assess value. This, in turn, allowed the inclusion of non-generic drugs for the first time if the cancer indication was a disease with high prevalence in the population and the benefit was cure or near cure. Dr. Yu concluded by stating that overall none of the current value models are perfect or necessarily easy to use. However, these models serve as a starting point to engage discussion and may be used to support, or develop a new value framework for immunotherapy.

**Table 1:** Current Value Frameworks

Framework	Factors considered	Purpose	Costs measured?	Perspective
ASCO Framework	Net Health Benefit: Clinical Benefit (OS > PFS > RR), Toxicity, Extended Survival	Comparison of two regimens that have been reported in a randomized clinical trial	No	Patient
ESMO-MCBS	Magnitude of Clinical Benefit: Prognosis of Condition, Clinical Benefit (OS, PFS), Long-term Survival (HR, RR), Quality of Life, Toxicity	Comparison of magnitude of benefit of regimens with reported comparative research outcomes	No	Societal
NCCN Evidence Blocks	Efficacy, Safety, Quality of Evidence, Consistency of Evidence, Affordability	Visual representation of key factors that provide information about the value of the recommendations within the guidelines	Yes	Patient
MSKCC Drug Abacus	Efficacy, Cost Toxicity, Treatment Novelty, Costs of Development, Rarity of Disease, Population Burden of Condition	Intended to provide information regarding the proper pricing of new drugs in the market	Yes	Societal
ICER Value Framework	Incremental Cost - Effectiveness Ratio: $\frac{\mathrm{Cos}{\mathrm{t}}_{\mathrm{new}}-\mathrm{Cos}{\mathrm{t}}_{\mathrm{s}\mathrm{tandard}}}{\mathrm{Effectivenes}{\mathrm{s}}_{\mathrm{new}}-\mathrm{Effectivenes}{\mathrm{s}}_{\mathrm{s}\mathrm{tandard}}}$	Comparison of two treatments based on efficacy and cost	Yes	Societal

*Abbreviations*: *OS* Overall survival, *PFS* Progression-free survival, *HR* Hazard ratio, *RR* Response rate

#### Cancer immunotherapy perspective on current value models

Continuing the discussion on how well current value frameworks capture the unique aspects of cancer immunotherapies, Dr. Michael B. Atkins provided an overview of the biological principles and clinical characteristics that make immunotherapeutics unique compared with convention chemotherapy and tumor-targeted therapies. Because immune-based agents activate a pluripotent immune system rather than inhibiting individual pathways within cancer cells, cancer immunotherapies mediate anti-tumor activity indirectly. The most important difference between conventional tumor cell directed therapy and immunotherapy is the potential for the immune system, when optimally activated, to eradicate all tumor cells. This effect is associated with durable responses in selected patients that are sustained off treatment, leading to apparent cure of some patients with metastatic disease. This phenomenon was initially reported when durable responses were observed in early studies using high-dose interleukin-2 (HD IL-2) to treat patients with melanoma and kidney cancer [7, 8]. The hallmark of immunotherapy demonstrated by these early studies is the long-term benefit experienced by a proportion of patients, and this can be mathematically represented by the flattening of the tail end of the Kaplan-Meier survival curve as demonstrated in long-term follow-up of patients treated with HD IL-2. In recent years, the proportion of melanoma patients experiencing durable responses has increased from 5–10% with HD IL-2 to 20–22% with ipilimumab treatment, 35–40% with anti-PD-1 agents, and has the potential to rise to upwards of 50% with the combination or ipilimumab and nivolumab [9]. Further advances using combinatorial immunotherapy approaches as well as developments in biomarkers to select patients for treatment have the potential to greatly increase the proportion of patients experiencing durable responses from these agents. For example, reports using ipilimumab and nivolumab in NSCLC have already shown efficacy as high as 92% in PD-L1 positive populations [10].

Given the potential of long-term survival following treatment with immunotherapy, Dr. Atkins illustrated that the current value models are overestimating the costs of these agents and not accurately representing their overall value. In particular, the current value frameworks fail to measure the characteristic treatment-free tail end of survival curves, and therefore, the potential for achieving long-term survival associated with immunotherapy treatment. Dr. Atkins emphasized that the costs of immunotherapies should be most accurately amortized over the longer horizon of benefit in a “cure-rate” model. Current value models may also overestimate the toxicities of immunotherapy. Although immunotherapeutic approaches can have high rates of drug-related adverse events (AEs), these side effects are often manageable and can be resolved quickly if recognized early and managed appropriately. Even in the context of combination approaches, previous studies have shown that 80% of AEs resolve in 4 to 6 weeks with immune-modulating interventions, such as corticosteroid administration, and that very few treatment-related deaths have been reported [9, 11]. Importantly, the management of these side effects doesn’t appear to interfere with therapeutic activity. Another important distinction is the extended treatment-free survival that patients can experience with cancer immunotherapy, which reduces the cost, inconvenience and overall toxicity of therapy, relative to non-curative approaches, and most importantly allows patients to return to their normal lives. This includes returning to productive work, contributing to their family’s well-being and their community, and having the opportunity to travel and be present for important life milestones. The positive effects of immunotherapy on a patient’s quality of life, as well as that of their family and communities, have not been captured by existing value frameworks. In addition, the current value models underestimate the benefits of immunotherapy for society, with the annual benefit of curing just 1% more patients with cancer estimated at $500 billion [12]. The models also do not consider the potential reduction in additional treatment for patients who respond to immunotherapy and will not require other forms of subsequent therapy. The cure-rate model also has its limitations since this favors short-term, high expenditures based on an anticipated savings in the future. This may further potentiate inherent challenges with respect to sustainability in the current healthcare fiscal environment. Economic models can be developed that adjust the pricing or cost of therapy to account for the fact that the benefits are accrued over time and not necessarily immediately.

Dr. Atkins also presented a variety of ways in in which the costs of immunotherapies could be reduced. Because many clinical trials empirically treated patients for a prolonged time (e.g., 1–2 years) and the kinetics of radiologic response to immunotherapy may lag well beyond actual tumor response, patients may be treated longer than necessary with current immunotherapies. To illustrate his point, Dr. Atkins stated that his group has been successful in stopping treatment without relapse even in patients with residual abnormalities on CT scans, suggesting that many of the residual abnormalities do not represent viable tumor. In addition, Dr. Atkins pointed out that immunotherapy combinations may actually be less expensive than single agents if they work faster, and therefore, require less total drug to be administered. Other ways to cut costs include avoiding combination approaches that focus on enhancing the benefit of non-curative treatment approaches rather than the immune effect, and therefore, don’t allow for cessation of treatment. In addition, biomarkers should be used to help guide the optimal immunotherapy combination for a particular patient. He predicted that because of the broad activity and high success rate of checkpoint inhibitor trials, the average cost of bringing a drug to market will decrease and the number of drugs available will increase resulting in more market competition. These effects are anticipated to help lower treatment costs. Overall, Dr. Atkins summarized that the current value models overestimate the costs of cancer immunotherapy treatment, overestimate the impact of acute but reversible toxicities, and underestimate the benefits of long-term as well as treatment-free survival, leading to a significant underestimation of the value of these therapies.

#### Economic outcomes of cancer therapy and the ISPOR initiative on U.S. value assessment frameworks

Dr. Lou Garrison offered insights from an economic perspective, including an overview of the definition of value, emerging value frameworks, and details concerning the International Society for Pharmacoeconomics and Outcomes Research (ISPOR) Initiative on U.S. Value Assessment Frameworks. From an economic perspective, value can be defined by what someone is willing to pay for or forgo to obtain something, which is variable across individuals as well as over time. Thus, it is difficult to measure in healthcare, where decisions are made behind the veil of insurance, even though cancer may be generally accepted as a potentially fatal disease requiring higher costs to attain quality outcomes. In defining the economic value for a particular treatment, the key drivers typically considered are: (a) health gain in terms of mortality reductions (i.e., resulting in life years gained), (b) any cost offsets (to drug price) due to reductions in the use of other health care, (c) improvements in morbidity and quality of life, and (d) the price of intervention. Dr. Garrison highlighted that in order to determine improvements in quality of life, patient engagement is critical to identify the important attributes of a specific disease. Within value frameworks for healthcare, Dr. Garrison also emphasized that concepts such as “value” and “total costs” can be difficult to measure. For example, the marginal costs of making pharmaceutical agents is very low; however, the oft perceived “high” price paid to manufacturers also must reflect the reward for innovation to cover the cost associated with bringing drugs to the market. With regard to the unique aspects of immunotherapy, the core measures of economic value can be broadened to incorporate additional measures, including reduction in uncertainty about therapeutic benefit, improvements in population-level adherence and uptake, innovation that leads to other scientific advances, extended survival that creates options for future advances, and the value of hope in the potential for a cure. These issues have not been addressed for either cancer treatments in general or specifically for cancer immunotherapy.

Dr. Garrison also described some of the current value frameworks for oncology drugs in the U.S., pointing out that these models should be viewed as complementary to one another. These frameworks tend to focus on “shared decision-making” between the clinician and their patients. Although each of these frameworks has strengths and limitations based on its objectives, Dr. Garrison argued that the emergence of a variety of different frameworks has caused variability in the evaluation of therapies and uncertainty within the field. Therefore, the ISPOR Initiative on Value Assessment Frameworks was developed to promote the development and use of high-quality, unbiased value assessment frameworks. To do so, an expert Steering Committee was convened to identify and discuss the key methodological and process-related issues in defining and applying value frameworks. Based on this discussion, a special Task Force was assembled to engage key stakeholders to produce a policy white paper to review current value approaches, with the intention to provide guidance on the appropriate definition and use of high-quality value frameworks. The overall goal of this white paper, which is expected to be released in May of 2017, is to enable more efficient health sector decision-making in the U.S. Dr. Garrison concluded his presentation by describing the challenges and next steps to assessing value models, which includes identifying what and how to measure elements of value as well as determining decision-making rules to apply these measures.

#### Patient perspectives: tales from the tail end of the survival curve

Providing a more personal perspective in his presentation, Mr. Silverstein illustrated the importance of incorporating patient perspectives when defining and prioritizing measures of value. As chairman of the board for the Melanoma Research Foundation and a melanoma survivor after receiving immunotherapy with HD IL-2 13 years ago, Mr. Silverstein provided a unique view of the value of immunotherapy through his experience as a patient. In his presentation, Mr. Silverstein considered the overall cost of multiple courses of treatment, including surgery, radiation therapy, and chemotherapy combined with the ongoing distress and uncertainty for the patients receiving these treatments as well as their families. Although the financial costs of HD IL-2 were high with multiple hospitalizations, no further therapy was needed after completing treatment. Emphasizing the potential societal impact of surviving cancer, Mr. Silverstein also described his feelings of gratefulness and desire to give back to the oncology research community. Through his roles on the board of trustees at local hospitals, chairman of the board of the Melanoma Research Foundation, and as a patient advocate on panels to review funding initiatives, Mr. Silverstein has worked to promote and lobby for increased funding for cancer research. With the increasing number of patients who are receiving and responding to immunotherapy, Mr. Silverstein challenged the audience to try to measure the value of the potential societal impact of these survivors. Since his initial diagnosis of stage IV melanoma, treatment with HD IL-2 has allowed Mr. Silverstein to travel with his wife, watch his daughters go through college and graduate school, and is now looking forward to celebrating their upcoming weddings. As an individual patient, he considered his immunotherapy treatment to be valuable, and concluding his presentation, Mr. Silverstein noted that he would “call that a really, really good value.”

#### Patient outcomes perspective: patient-reported outcomes and personal cost of cancer

Drs. Adam P. Dicker and Heather S. Jim jointly presented their perspective on the importance of incorporating patient-reported outcomes (PROs) in clinical trials as well as their ability to enhance current value models. PROs, or the status of a patients’ health reported directly from the patient, can be used to illustrate the clinical benefit, such as changes in disease-related symptoms. For example, in the Checkmate-025 trial, a phase III study of nivolumab versus everolimus in previously treated patients with advanced or metastatic renal cell carcinoma, investigators included a PRO measure of kidney cancer-specific symptomatology. In patients taking nivolumab, symptom improvement was shown at 4 weeks that continued through 2 years of follow-up, illustrating that nivolumab may improve the quality of survival, in addition to the time-based overall survival benefit reported [13]. In value models, PROs are very important to evaluate toxicities and can help model the costs associated with managing them [14]. Standard measurements of AEs used in clinical trials have been shown to inaccurately capture toxicities, with reporting sensitivities at less than 50% for some AEs [15, 16].

PROs can also help improve system management, which can result in improved outcomes in cancer patients. In particular, a recent report illustrated that clinic-based monitoring of 12 common symptoms using the PRO-Common Terminology Criteria for Adverse Events (PRO-CTCAE) improved quality of life, reduced emergency room visits, increased the median number of months on chemotherapy, and increased quality-adjusted 1 year survival rates among cancer patients [17]. Interestingly, there were also more pronounced benefits for computer-inexperienced patients who used electronic software to report symptoms, suggesting a role for PROs to address heath disparities [17].

Recent technological advances in collecting PROs were also presented to address deficiencies of current measures, including failure to report AEs during gaps in treatment visits and the overall underreporting of symptoms. Smart phone-based applications have been developed to provide real-time monitoring of PROs. In addition, personal technology monitors can now also be utilized to captures measures such as sleep, activity level, and basic vital signs. This enhanced PRO monitoring can be used to improve monitoring to more accurately capture clinical benefit as well as the management of treatment side effects, which can in turn enhance shared decision making, affect regulatory approval of drugs, and could be used to help determine the value of different therapeutics [18]. Dr. Dicker concluded with a call to action, outlining the need to develop common PRO measures, particularly in immunotherapy clinical trials, and to subsequently incorporate these measures into routine clinical care.

#### Session I panel discussion

The first session of the symposium concluded with a panel discussion with audience questions moderated by Dr. Howard L. Kaufman. Highlights of this discussion included whether improvements in current frameworks would be sufficient to address the weaknesses identified in current value models. Overall, it was determined that current models need to incorporate further measures to make them more applicable to modern immunotherapy regimens. Among the suggestions discussed, increasing the role of the patient and PROs in determining value and an improved effort to collect post-marketing outcome data, were considered important. The rapid progress in tumor immunotherapy will require integration of such emerging data as it becomes available to fully define the value of immunotherapy agents and combination regimens. In addition, the importance of engaging patient advocacy organizations in the discussion of value was emphasized, to also bring the disease-specific issues for different cancers to the value framework discussion. The participants also acknowledged that additional issues, such as financial toxicity for patients required to make increased co-payments and the potential for conflicts of interest between industry funded patient assistance programs and not-for-profit patient advocacy foundations, may also require additional discussion.

### Session II: Other perspectives on the value of cancer immunotherapy

#### Payer perspective

Opening the second session, Dr. William McGivney provided payer perspectives based on his experiences as a Vice-President for Coverage at a major national payer, his membership on the Medicare Coverage Advisory Committee (MCAC), and working as a consultant in the biopharma industry. He began his presentation with insights on how value is viewed from the payer perspective and by providing an overview of what it’s like to be a payer in the current healthcare climate. In his introduction, Dr. McGivney presented the concerns of insurance providers with expensive specialty pharmaceuticals in oncology, including immunotherapy agents, and highlighted the fears around the potential of combination approaches utilizing multiple costly agents. Essential to the discussion on value is also an understanding of the complexity of the contemporary healthcare environment, which consists of multiple organizations driving clinical decision-making as well as value metrics and multiple payment methodologies that insurance companies must take into consideration in a changing health care delivery system. Within this complicated environment, some insurers would like to be more of a service company that sells, for example, utilization management services like precertification, formulary management, etc. to large employer customers. Increasingly and ironically, insurance companies (i.e., payers) are seeking to offload risk via new payment methods for providers and outcome-based risk sharing arrangements with biopharma companies. Insurance companies must also ensure that they remain competitive and “cutting edge” in regard to the plans that they sell to employers in the marketplace.

Historically, insurers have been apprehensive about having strict utilization management practices for oncology drugs/biologics (e.g., precertification, step therapy, formulary exclusion, etc.). However, with the development of an increasing number of agents, payers are exerting more scrutiny and inserting themselves into coverage policy decision-making to actively manage oncology therapeutics. In particular, as multiple agents with the same or similar mechanisms of action are approved within the same indication (e.g., multiple agents inhibiting the PD-1/PD-L1 pathway), payers will have more leverage to initiate preferred agent status and to look for opportunities for discount arrangements with pharmaceutical companies. Overall, payers are somewhat interested in risk-sharing and subsequent risk-based contracts, but there is uncertainty as to how to implement these agreements in such a complex, ever-changing environment.

Dr. McGivney also provided insight about the deficiencies of the current value frameworks, again highlighting the need for increased patient involvement in the development of these models. In particular, he also warned about the deficiencies of ICER model based upon its derivation from and use within the National Institute for Health and Care Excellence (NICE) in the U.K., as it has been associated with poorer outcomes and lower overall survival rates across multiple tumor types for the U.K. compared to other developed Western European countries [19]. It is possible that the lower survival in the U.K. may be explained by a diagnostic time bias in the most common cancers, while the U.K. could be considered more efficient with better heath indicators at the population level resulting in lower overall health expenditure when compared to the U.S. Moreover, the report issued by ICER on drug value in NSCLC was recently called into question based on its ability to interpret clinical evidence and reach conclusions based on measures that are scientifically rigorous, comprehensive in scope, and unbiased in nature [20].

In the final portion of his presentation, Dr. McGivney presented the current landscape and potential direction of alternative payment models. The overall objective of CMS is to move away from a fee-for-service approach toward the Medicare Incentive Payment System (MIPS) that will provide bonuses or penalties (2019) based upon provider performance. The more financially risky CMS bundled payment program is also being implemented. In this program, providers may assume more downside risk in anticipation that through tight management their practices can reap increased payments based upon the difference between practice treatment costs and the assigned bundled payment. However, to change the healthcare system from a fee-for-service model while still ensuring that there is adequate coverage of services, appropriate compensation for clinicians, and access for patients is a very difficult task. Dr. McGivney concluded his presentation by highlighting the complexity of developing coverage policies that have the potential to affect such diverse populations of stakeholders. In addition, Dr. McGivney challenged policy makers and stakeholders to consider how decisions based upon the policies and processes implemented may affect individual patients and their families. Importantly, he suggested that payers should have more empathy by considering the possibility that their decisions directly impact access to care for patients and may affect patient’s family and friends as well as payer’s loved ones. As such, these decisions must be directed at meeting the needs of the patient whose treatment recommendations are being considered.

#### Industry perspective

Dr. Jon M. Wigginton presented broad perspectives from industry on the value of cancer immunotherapy. In the introduction of his presentation, Dr. Wigginton provided a brief history of the cancer immunotherapy field, spanning the initial findings using Coley’s toxin to the modern day success of T cell checkpoint inhibitors to the promise of chimeric antigen receptor (CAR) T cell therapies. Although historically these agents have been thought of as toxic, complex, and having limited clinical activity, the recent progress in the field has resulted in an era where cancer immunotherapy now needs a distinct value proposition [21–23]. Dr. Wigginton suggested that the findings in the randomized study of ipilimumab in patients with metastatic melanoma published in 2010 was a turning point in the cancer immunotherapy field [22]. As the first study using an immunotherapy agent to show significant prolongation of overall survival, this study also demonstrated that a short course of immunotherapy and a relatively low rate of objective response could translate into meaningful clinical benefit. Moreover, this study also illustrated the unique patterns of clinical response and the unique aspects of immune-related adverse events [24]. Further reports using immune checkpoint inhibitors targeting the PD-1/PD-L1 pathway underscored the significance of these findings and illustrated additional data to support the durable, immune-mediated patterns of response with T cell checkpoint blockade [25–27]. Based on these foundational studies, checkpoint inhibitors now have indications in a variety of disease settings and are likely to become the backbone for future combinatorial approaches. In highlighting the unique aspects of immunotherapy, Dr. Wigginton emphasized the need to consider the value of the tail end of the Kaplan Meier survival curve that is characteristic of immunotherapy.

A variety of issues from an industry perspective were also introduced that pertain to the discussion on value. Some of the questions/challenges posed by Dr. Wigginton included how to effectively capture the unique aspects of clinical benefit, how to address the unique challenges in developing immunotherapy agents, how to effectively capture the patient experiences outside of conventional evaluation criteria, and how to reconcile the cost of innovation vs. value in the context of agents with broad potential use. In order to address these challenges, Dr. Wigginton emphasized the need for a patient-centric model to maximize patient value. In addition, within this model the cost of innovation must also be sustained and complexities in the reimbursement structure should be considered. Current value frameworks are limited in several areas, including the lack of collaboration in their development, lack of applicability to immune-based agents, lack of long-term quality of life measures, focus on drug costs vs. the total cost of care, and a disconnect between pharmacy drug budgets and resources within healthcare systems. Dr. Wigginton concluded his presentation with an overview of the promise as well as challenges for developing biomarkers to predict and monitor response to immunotherapy agents. In order to overcome the challenges associated with biomarker development, he proposed the idea of generating a broad-based, “Manhattan-project” style research collaboration across the immuno-oncology field to identify potential biomarkers for cancer immunotherapy.

#### Predictive and companion biomarkers

In the final presentation of the summit, Dr. Roy S. Herbst presented perspectives of predictive and companion biomarkers based on the recent developments in lung cancer. Dr. Herbst began his presentation with a 10-years history of the progress in the treatment of lung cancer, beginning with the development of targeted therapies and ending with the recent breakthroughs that resulted in first-line approval of pembrolizumab for NSCLC. The experience in lung cancer with precision medicine and the use of targeted therapy nicely illustrates the need to look both within and beyond tumors for predictive biomarkers. As a result of such biomarker-driven trials as the Lung-Map study, large numbers of patients are being screened to identify molecular markers to better optimize therapy. Currently, these large biomarker-driven trials are focused on therapies targeted to specific-driver mutations within tumors, while the patients who test negative for these mutations go on to receive immunotherapy. However, in the near future, this study design could be utilized to include immune-based biomarkers to test novel immunotherapy combinations.

Presenting results from the first early phase trial of nivolumab, Dr. Herbst concluded that immunotherapy is having a significant impact on patients with lung cancer. Dr. Herbst highlighted the recent breakthroughs in NSCLC that have led to the approval of nivolumab, pembrolizumab, and atezolizumab in the second-line setting as well as the recent approval of pembrolizumab frontline for patients with advanced NSCLC [28–31]. The seminal Keynote-024 study demonstrated significant progression-free and overall survival benefits for patients with advanced NSCLC after frontline treatment with pembrolizumab compared with platinum chemotherapy. Importantly, this study was also performed in a preselected population of patients whose tumors had a PD-L1 tumor proportion score (TPS) ≥ 50% [30]. This biomarker cutoff proved to be very important, as the CheckMate-026 study with a lower PD-L1 TPS cutoff at ≥ 5% failed to show improvements in survival for nivolumab in the first-line setting [32]. Recent data presented from combination approaches has also illustrated that PD-L1 has utility as a biomarker. In combination approaches using chemotherapy plus pembrolizumab, there were reasonable response rates even in the PD-L1 negative population, suggesting a potential role for chemotherapy to help drive an immune response. Moreover, PD-L1 high patients (TPS ≥ 50%) have response rates over 90% following treatment with frontline ipilimumab plus nivolumab treatment [10]. Although PD-L1 has proved to be a useful biomarker in certain settings, Dr. Herbst also described the limitations of this marker by presenting the potential variation in PD-L1 expression throughout a single tumor sample and the challenges of having several different assays, which has highlighted by the effort of the Blueprint Proposal to compare the three currently approved biomarker assays for PD-L1 expression [33].

Shifting the focus to the future of biomarkers as predictors and determinants of response to immunotherapy, Dr. Herbst presented work to illustrate the predictive value of measuring tumor infiltrating lymphocytes (TILs), mutational burden, and gene expression analysis signatures from his Lung Cancer SPORE Team at Yale University. In ongoing studies, markers to detect the presences of TILs with PD-L1 expression are being explored to help guide therapy for patients whose tumors are immunocompetent and to determine ways to illicit immune responses in tumors that do not currently show evidence of immune activation [34]. Another area of increasing interest is measuring mutational burden as a predictive marker, which was based on findings that illustrated a correlation between mutation burden and response to checkpoint inhibition [35]. In addition to mutational burden, gene expression analysis can be used to identify patients with “inflamed” tumors using specific immune-based signatures [36]. Concluding his presentation, Dr. Herbst emphasized that combination approaches will be key to the future of immunotherapy, and it will be important to look at a variety of different markers to better guide combination approaches as well as to identify mechanisms of resistance to guide patient treatment selection. Overall, identifying reliable biomarkers will improve efficacy, decrease toxicity, and therefore optimize the cost and value of these agents.

#### Session II panel discussion

Moderated by Dr. Atkins, the final extended panel discussion included additional panelists from a variety of pharmaceutical companies, including Kirsten Axelsen, MS (Pfizer), Daniel S. Chen, MD, PhD (Genentech), Ravinder Dhawan, PhD (Merck & Co), and Gregory Keenan, MD (AstraZeneca). In the opening of the panel session, the issue of when to stop immunotherapy and ongoing initiatives to address this issue were discussed. Several panelists confirmed that clinical trials were ongoing to investigate the timing and duration of treatment; however, due to the varying response patterns seen in previous studies, the necessary treatment duration for immunotherapy is still unclear at this point. It was also emphasized that post-marketing data is needed to better inform patients and their physicians who are fearful of stopping therapy, as extended treatment not only adds to the cost of therapy but may also contribute to delayed toxicities. Further discussion focused on the current state of biomarkers for both single-agent as well as combination approaches. The panelists confirmed that there is an enormous effort underway to identify novel biomarkers and to build on the current PD-L1 assays. It was agreed that biomarkers will likely play a large role in the value of cancer immunotherapy. The panel considered that if a validated biomarker was available, it would be used to direct treatment selection for particular patients, monitor responses during and after treatment, and help in determining mechanisms of resistance that could inform decisions about subsequent treatments as well as the development of optimal combination treatment approaches. Other issues discussed included the need for large collaborative projects to identify biomarkers on a larger scale and whether combination approaches should be used upfront to help combat subsequent emergence of treatment resistance. As a final conclusion to the summit, the panelists shared their thoughts on how to ensure patient access to new immunotherapy agents. The panel discussion highlighted the need for collaboration between key stakeholders, in particular patients, physicians, and policy makers, to prevent the development of unnecessary regulations and costs that would restrict access. In addition, panelists from industry highlighted efforts to increase patient access through discount and reimbursement programs. In this discussion, it was emphasized that to ensure patient access, the overall value of immunotherapy needs to be better established in order to justify the financial costs associated with these therapies. Other critical issues include sustainability and the additional costs of new diagnostic procedures applied at larger scales across larger patient populations.

## Conclusions

Dr. Atkins concluded the Summit by reflecting on the remarkable success of cancer immunotherapy in recent years. He then challenged the audience to continue to drive this success by striving to make cancer a “curable” rather than a “chronic” disease. Building on the outcomes of this Summit, SITC will continue to better define the value of cancer immunotherapy in order to inform all stakeholders of the unique properties of immunotherapy and to ensure that patients and healthcare professionals feel empowered to make informed treatment decisions about their care and have access to high-quality treatment implementation. A better definition of the value of cancer immunotherapy will also benefit society in helping to reduce oncology costs by promoting the development of new drugs and ensuring that patients with cancer have access to the most effective treatment. In addition, this will also help to limit potential adverse events and improve individual patient outcomes with cancer immunotherapy.
